# A Medical Causerie

**Published:** 1916-02-12

**Authors:** 


### A MEDICAL CAUSERIE.

### The Treatment of Wounds.

We do not hear very much of the actual effect produced on the practice of the R.A.M.C. by the controversy relative to the treatment of wounds. This silence is probably due more to disciplinary considerations than to lack of time. Whatever be the explanation, there has been but little indication to the profession of the clinical results of the attempt to replace the use of antiseptics by methods having a bacteriological basis. But it is quite certain that all is not smooth sailing. It is only necessary to read a recent article by Sir Watson Cheyne to realise that the surgeons are not all ready to yield to the bacteriologist. Sir Watson has clear convictions and a vigorous pen, and he prefers to call things by plain names. The "hypertonic salt solution" or "phylacagogic agent" becomes in his vocabulary "brine," with qualities competent to destroy all living things—provided only it is strong enough. The notion of gentlemen armed with capillary tubes promenading the surgical wards and ready to teach the surgeon when the psychological moment for interference has arrived, receives no welcome from Sir Watson Cheyne, who, indeed, has clearly made up his mind that these laboratory persons are getting somewhat out of their proper sphere. The suitable place for them, he suggests, is not France, but England. It is there lies their great opportunity, which is to set to work during the next six months to raise the immunity of the new recruits to such a point that whenever any of them is wounded he will either escape infection altogether or will be only slightly affected, and therefore amenable to treatment. As an incidental result of this course, the surgeons at the Front could take advantage of the absence of their laboratory colleagues to try what could be done by some purely surgical methods. All this goes to show the need for considerable freedom of opinion and action in medical questions, even when these are associated with official organisations. Indeed, such association heightens the need for personal liberty of decision because it tends to suppress initiative. What the chiefs of an organisation almost invariably wish is smooth and quiet working, and an officer who "stirs things up" is more likely to get snubbed than promoted. The fixed rule is so delightful, for it saves everyone the trouble of thinking. All this is not the atmosphere in which scientific progress is likely to occur, and while there must in practice be some check to individual vagaries and "cussedness," it will be a bad day for the Army when each medical officer directs his treatment, not by his individual judgment, but according to a scheme imposed from above.

### Deaths in Action.

The sad tale of deaths in action or from wounds continues, and the medical profession takes its share with the rest of the community. The names of some of our young *confrères* are added every week to the Roll of Honour. It seems right that an effort should be made to preserve the memory of these men. Photographs with short biographical notices often appear in medical school journals or in local newspapers, and it would need no great effort to collect these in the hope that at the end of the war they could be published in a volume which would surely appeal to every member of the profession. It will be seemly that in every medical school and at every medical social gathering there shall be, at the restoration of peace, some due recognition and honour paid to the memory of those members of the profession who have died in the great war and for the good cause.

### A Gratuitous Insult.

There must be a number of active commercial minds who regard doctors as very simple-minded persons. At least this conclusion would seem to be prompted by some of the advertising schemes submitted to the profession. A little time ago practitioners were offered, on application, some kind of pocket-pen which would remind the user of the virtues of an infants' food or a tonic syrup. The proposition hardly complimented the profession on the point of dignity, but, after all, a pen is not a very serious matter, and if an advertiser likes to thrust one into a person's pocket many might say the matter is not conspicuously objectionable. But surely the line is passed when some stranger, titled or otherwise, writes to invite you to spend a week-end at his hotel "somewhere," let us say, "on the sea front." Whether the hospitable host attends in person to see to the comfort and entertainment of his guests must be left an open question, but it is very difficult to think of any self-respecting person getting any pleasure out of such an invitation. And there are those who think the whole proceeding is an example of presumption and impertinence, so that its issue, instead of being "good business," may prove rather a harmful speculation. The gentry who think that recommendations from the profession are to be bought by such proceedings are not to be encouraged. The hotel here in question may be everything which is delightful, but some members of the profession will take care to give it a wide berth. Happily, the profession in this country holds to the view that in all such matters it is impossible to be too strict. The desirable thing is that the practitioner, in dealing with his patient, shall have a judgment which is entirely detached from selfish motives, and to secure this it is necessary to avoid even the appearance of evil. Take the present instance. A practitioner accepts the invitation, and later on recommends one or more patients to the hotel. Let these know that the doctor got a free week-end, and it is easy to imagine what will be their opinion both on himself and on his advice.

**The Scottish War Service Committee.**

The statement made in the Medical Causerie of January 29 relative to the origin of the Scottish War Service Committee needs a little modification. It is true that the Medical Corporations have from the outset been represented on the Committee by their Presidents, and that they have in other ways given assistance and support. The same, too, has to be said of the Medical Faculties of the Universities. But it seems that the initial step which led to the formation of the Committee was taken by Dr. Hamilton, the Chairman of the Scottish Committee of the British Medical Association, and hence it was hardly correct to say that the credit for this was due to the Corporations. The practical point is that from the outset there was recognised the necessity of a full representation of all interests, and hence the Committee has never incurred the suspicion of sectional aims or motives. Both in the hour and in the fashion of its birth it was more happy than its English correlative, for, reasonably or otherwise, there is in the South a certain section of the profession which will not believe that any good thing can come out of the British Medical Association Nazareth. Apart from all question of its constitution the Scottish Committee has fully secured the confidence of the profession by the thoroughness and tactfulness which have characterised its work, and, indeed, its lead was the stimulus which aroused action in the South. Much credit is due to the Convener, Dr. Norman Walker, and there is general recognition of the services rendered by Dr. J. R. Currie, whose organising abilities have been most valuable. To come back to the starting-point of this note, the Committee has long since achieved such a position that no one asks as to its origin; it is accepted as a piece of machinery admirably adapted to deal with the special professional emergencies of the hour, and if its true begetter does not always receive the credit which is his due, he must be content to believe that the word he uttered was already on the tongue of the rest of his countrymen.

